# An analysis of the adaptability of a professional development program in public health: results from the ALPS Study

**DOI:** 10.1186/s12913-015-0903-3

**Published:** 2015-06-14

**Authors:** Lucie Richard, Sara Torres, Marie-Claude Tremblay, François Chiocchio, Éric Litvak, Laurence Fortin-Pellerin, Nicole Beaudet

**Affiliations:** IRSPUM, Université de Montréal, P.O. Box 6128, Centre-ville Station, Montréal, QC H3C 3 J7 Canada; Faculty of Nursing, Université de Montréal, Montréal, Canada; Léa-Roback Research Centre on Social Inequalities of Health in Montréal, Université de Montréal, Montréal, Canada; Department of Family Medicine, McGill University, 5858 Côte-des-Neiges Road, 3rd floor, Montreal, QC H3S 1Z1 Canada; Telfer School of Management, University of Ottawa, 55 Laurier Ave. East, Ottawa, ON K1N 6 N5 Canada; Institut de recherche de l’Hôpital Montfort, Ottawa, ON Canada; Public Health Directorate for Montreal, Montreal Health and Social Services Agency, 1301 Sherbrooke St. East, Montreal, QC H2L 1 M3 Canada; Department of Social and Preventive Medicine, Université de Montréal, Montréal, Canada

## Abstract

**Background:**

Professional development is a key component of effective public health infrastructures. To be successful, professional development programs in public health and health promotion must adapt to practitioners’ complex real-world practice settings while preserving the core components of those programs’ models and theoretical bases. An appropriate balance must be struck between implementation fidelity, defined as respecting the core nature of the program that underlies its effects, and adaptability to context to maximize benefit in specific situations. This article presents a professional development pilot program, the Health Promotion Laboratory (HPL), and analyzes how it was adapted to three different settings while preserving its core components. An exploratory analysis was also conducted to identify team and contextual factors that might have been at play in the emergence of implementation profiles in each site.

**Methods:**

This paper describes the program, its core components and adaptive features, along with three implementation experiences in local public health teams in Quebec, Canada. For each setting, documentary sources were analyzed to trace the implementation of activities, including temporal patterns throughout the project for each program component*.* Information about teams and their contexts/settings was obtained through documentary analysis and semi-structured interviews with HPL participants, colleagues and managers from each organization.

**Results:**

While each team developed a unique pattern of implementing the activities, all the program’s core components were implemented. Differences of implementation were observed in terms of numbers and percentages of activities related to different components of the program as well as in the patterns of activities across time. It is plausible that organizational characteristics influencing, for example, work schedule flexibility or learning culture might have played a role in the HPL implementation process.

**Conclusions:**

This paper shows how a professional development program model can be adapted to different contexts while preserving its core components. Capturing the heterogeneity of the intervention’s exposure, as was done here, will make possible in-depth impact analyses involving, for example, the testing of program–context interactions to identify program outcomes predictors. Such work is essential to advance knowledge on the action mechanisms of professional development programs.

**Electronic supplementary material:**

The online version of this article (doi:10.1186/s12913-015-0903-3) contains supplementary material, which is available to authorized users.

## Background

Public health practice is constantly evolving, shifting frequently as new discourses emerge across the field and the practice context is transformed by health system reforms [1, 2]. Professional development, broadly conceived as a conscious effort to enhance professionals’ learning [3, 4], is considered a potential lever for action to support these changes [5–7]. However, the training, experiences, practice settings, and continuing education needs of the public health workforce are extensive and varied, complicating professional development efforts [8]. This variability calls for adaptive professional development programs that can meet the needs of a broad spectrum of professionals in different settings. Traditionally, educational and training programs have been developed linearly through a process in which they are designed, tested, and implemented using manualized protocols to minimize deviations from the original model and with little consideration for the implementation context [9, 10]. While faithful to intervention theory, professional development programs produced in this perspective often take the form of episodic and didactic information delivery, disconnected from professionals’ real-world experiences [11]. Unfortunately, “this decontextualization essentially disregards the value of ongoing and situated learning, thereby reinforcing the perceived divide between theory (what you learn in a course) and practice (what you do at work every day)” (p. 703) [11]. To be effective, professional development programs must be adaptable to different real-world practice settings while preserving the core components of their model.

Based on recent developments in complexity theories and planned adaptation models [10], this article reports results of an implementation evaluation of a professional development pilot program, the Health Promotion Laboratory (HPL). Our objectives in this article are: 1) to present the HPL, its core components and potentially adaptive features; 2) to analyze how it was implemented in three different settings; and 3) to explore the relationships between teams’ characteristics and contexts, on one hand, and the implementation profile in each site, on the other.

### Fidelity and adaptability of professional development programs

Empirical research has demonstrated that, to be effective, professional development must be contextualized, related to practice, and situated within a community that supports learning, rather than delivered in a discrete package using a didactic and disembodied approach [11–13]. This is especially pertinent in health promotion, where action is mostly contextual, community-based, and collaborative [14]. Conceiving of professional development as a social activity anchored in a practice environment necessarily entails designing programs that can be adapted to fit the diversity of public health and health promotion practice settings and that promote engagement with authentic work experiences in a multitude of ways [15, 11]. In fact, while a program’s core model and educational basis must not be neglected, the implementation cannot overlook the program’s delivery contexts, if the intervention is to be effective [9, 16]. An appropriate balance must be struck between implementation **fidelity**, defined as respecting the core nature of the program that underlies its effects, and **adaptability** to a specific context, to maximize the benefit of the intervention in a specific situation [9, 10, 16, 17]. In this paper, adaptation is conceived as the process of making a program fit a specific context or setting either by modifying it (for instance by reordering, delaying, or emphasizing components), changing the manner or intensity of delivering the program components, or adapting the program to cultural or language sensibilities [10, 16, 18].

Over the past decade, implementation science has proposed some guidelines to deal with the ‘adaptability–fidelity’ tension when implementing an evidence-based program in different settings. Indeed, given the impossibility of standardizing programs across different social and complex settings, some authors have proposed to focus on the integrity (fidelity) of the program and its core components [10, 16, 18]. Core components are those that account for the program’s effects, according to its theory, logic model, or empirical evidence [10, 16]. These core components often lie in the program’s theory (or change theory), which stipulates the program’s causal mechanisms [19]. According to this view, core components designed to trigger causal mechanisms must be implemented with fidelity to preserve program effectiveness, but secondary aspects of the program can be modified [10]. Likewise, Hawe et al. [15] propose redefining the concept of standardization by focusing on the various steps in the program’s change process and their key functions, rather than on structural and format elements of the intervention. This means the standard (or core) program components to be preserved in the implementation would be the various steps in the change process, or the objectives they are intended to facilitate. The intervention’s composition and format could then take on different forms to adapt to local contexts, while achieving the same objectives. “Context level adaptation does not have to mean that the integrity of what is being evaluated across multiple sites is lost. Integrity defined functionally, rather than compositionally, is the key” (p. 1562) [15].

In recent years, many authors have called for the development of a ‘science of replication’ [16, 17]. In this movement, authors such as Lee et al. [10] have suggested rational and planned approaches to adapt programs to different contexts. Arising from the need to implement evidence-based programs effectively in varied settings, the Planned Adaptation approach is presented as a “guide for adapting theory-based [programs] that directs practitioners to consider how population differences may relate to the content of program activities and the theory of change” (p. 291). The Planned Adaptation approach involves four phases: (1) examining the program’s theory of change; (2) identifying population differences; (3) adapting the program’s content; and (4) adapting the evaluation strategy. The first phase involves making explicit the core elements of a program’s theory that are critical to achieving its intended outcomes. The other phases broadly involve examining the implementation’s contextual characteristics and analyzing how they will interact with the causal mechanisms postulated by the program (phase 2) and then making the appropriate modifications to the program and evaluation strategies (phases 3 and 4). While this model proposes a straightforward and explicit approach to adaptation, it has the shortcoming of not taking into account the dynamic and complex nature of implementation contexts. Indeed, according to complexity theory, a program’s introduction into a social context can be seen as a critical event in a complex system, generating new emerging properties and a self-reorganization of the system [15, 20, 21]. This complexity also calls for ongoing adaptation of the program to the local context in its implementation [15, 22]. Indeed, Riley et al. [22], interested in the best practices for designing interventions in complex contexts, specifically point out the importance of tailoring the features of intervention strategies to the implementation context (achieving adequate implementation), but also of continuously adapting the intervention to the local environment while maintaining the integrity of the intervention (modifying interventions during implementation). In the next section we present how the HPL professional development program can accommodate these two kinds of adaptation, i.e., initial tailoring to the local implementation context and ongoing adaptation to participants’ needs.

### Background: The Health Promotion Laboratory

In Quebec, healthcare and public health are managed at three levels: *provincial*, governed by the Ministry of Health and Social Services; *regional*, administered by regional health and social services agencies; and *local*, delivered by health and social services centers, or CSSS (*centres de santé et de services sociaux*). The CSSS structure was created as part of the province’s 2004 health reform [23], when 95 CSSSs were created in Quebec, including 12 in Montreal. The mandate of these new organizations is to integrate the public health and the healthcare sectors using a population-based approach across a continuum of services ranging from health promotion all the way to palliative care [23]. Incorporating these new activities into professionals’ practices did not go smoothly [24, 25]. For example, a study of nursing practices in Montreal CSSSs after the reform showed that these were still oriented toward clinical care and individual preventive interventions, excluding community development and action on socio-environmental determinants of health [26]. In 2009, in response to these gaps, the Public Health Directorate for Montreal (PHDM) developed the Health Promotion Laboratory (HPL), a professional development program targeting multidisciplinary public health teams from CSSSs. Starting in 2010, the HPL was gradually implemented in partnership with seven CSSSs in the Montreal region.

### The program, its core components and adaptive features

The HPL offers an approach to professional development based on competencies development, reflective practice, and problem-based learning. Broadly speaking, the program supports a team of multidisciplinary practitioners in using a population-based approach to design and implement a specific health promotion intervention [27, 28]. More specifically, the program pursues synergistic objectives at three levels. At the individual level, participants are expected to develop new professional competencies in health promotion, as well as reflexivity with regard to their professional experience. Building on these new competencies, members of the team are expected to collectively develop and implement a new health promotion intervention targeting a specific issue (e.g. occupational health, student retention, children’s vulnerability). It is expected that new and improved team work processes, especially with regard to health promotion program planning, will be developed and gather strength through this experience. Finally, it is assumed that the new knowledge and practices will have a ripple effect at the organizational level through knowledge diffusion to colleagues and units not formally involved in the HPL.

Concretely, each HPL’s issue or theme is chosen by the participating CSSS based on its priorities. Once the broad theme is chosen by the organization, a team of voluntary participants is created to work on it in the HPL. Teams are composed of seven to ten professionals from various disciplines and managers. The HPL formula involves an ongoing and lengthy process in which participants meet for three hours every two or three weeks for 18 to 36 months, depending on the organization’s needs and preferences. Meetings are held during the participants’ normal working hours, and they are freed up by the organization to attend. Sessions are led by one of the participating managers or professionals, supported by a mentor from the program promoter agency (PHDM). Each team is guided by a different mentor but all follow the approach described below. External participants may be invited once in a while, for example, to provide key expertise or to liaise with partners from the organization. Mentors encourage reflection and discussion, offer feedback to participants, and guide the HPL teams by providing recommendations on processes and resources for learning, but the teams assume leadership. A detailed description of the HPL program is available elsewhere [27].

While the program does not build on one specific theory, it has been inspired by literature on competencies development, adult learning, reflective practice and organizational change [29–32]. It also capitalizes on the experiential knowledge of the program’s promoters. The HPL’s intervention theory (logic model), which formalizes the links between the resources, activities, and objectives of production [33], has been evaluated in a previous study using logic analysis [27]. Logic analysis is a type of theory-based evaluation by which the plausibility of a program’s theory can be tested based on available scientific knowledge [33, 34]. Logic analysis demonstrated that the program’s intervention theory had great potential for achieving the intended results.

The PHDM promoters developed an *operational approach* to guide the HPL activities. This approach, which stems from the program’s intervention theory, comprises seven steps that can be conceived as interrelated steps of a change process, each functionally defined by an objective (which corresponds to an objective of production). While each objective specifies a direction to take, the composition (content, activities, sequence) of each step is highly flexible, to be adapted to each team’s situation and needs. Also, the whole approach is iterative, and the form the overall process adopts may vary. The steps of the operational approach thus represent the program’s core components. Logic analysis confirmed the different steps of the operational approach as crucial components of the program [27]. In sum, while the steps of the operational approach represent the program’s core components, how they are implemented (format and number of activities, sequence of implementation) constitutes the adaptive part of the program. The program can be adapted at two levels: (1) before the implementation *per se*, with initial tailoring to the needs of the participating CSSS (issue selection, team composition, HPL formula); and (2) during the implementation, with ongoing adjustment to the different needs of the teams, which assume the leadership role in implementing the different steps of the HPL (sequence, content, activities).

Table 1 presents the seven steps of the operational approach. Each step’s possible activities are also presented, but these are examples and do not constitute a mandatory checklist.

**Table 1:** The seven steps of the HPL’s operational approach and examples of activities

Steps (core program components)	Examples of activities
*1) Identify a targeted issue and a team*	PHDM promoters introduce the HPL to the management and participants at a CSSS site. Management, having agreed to participate, has chosen a team interested in the HPL and selected a theme or targeted issue to work on.
*2) Specify the operational approach*	PHDM promoters and facilitators introduce participants to each step of the operational approach. Participants learn how the HPL came about and are informed of the issue they will work on, and of the CSSS and PHDM support available to implement the HPL. Each team fine-tunes the operational approach according to its needs. Teams usually strike a committee to plan and facilitate HPL meetings.
*3a)* ^a^ *Acquire basic concepts of public health and provide opportunities for reflection*	Participants acquire key concepts of public health and reflect on how these apply to their practice. Core training mechanisms include collective reading, reflection, and discussion of materials selected by PHDM promoters or participants.
*3b)* ^a^ *Transfer new knowledge and ensure sustainability of the program*	Team members discuss the professional development program and transfer the knowledge they have gained to other publics. Another objective in this step is to obtain buy-in and support for adoption and replication of the model from other staff in their division and upper levels of decision-making at the CSSS. To pursue these objectives, participants might learn how to write articles and make public presentations about their work on the HPL.
4) *Study the problem (theme or targeted issue)*	The team gains in-depth knowledge on the targeted issue assigned to them. Participants collect, analyze, and interpret data to develop a clearer picture of the issue. Activities include theoretical discussions about key concepts related to the issue, presentations by experts in the subject area, and practical exposure to clients’ needs through field visits in their territories.
5) *Identify options for action*	Participants discuss potential health promotion interventions to target the issue and decide collectively what strategies and actions to develop. Consulting the available literature and experts in the field are examples of activities for this step.
6) *Develop partnerships*	The team sets up a partnership with community stakeholders to be involved in the health promotion intervention. Activities may include weighing the advantages of collaborative action versus sectorialized action, identifying existing partners working in the territory, and creating new networks.
7) *Implement a new health promotion action or improve a current intervention*	Participants collectively plan the implementation of the intervention (or the improvement of a current intervention) to address the targeted issue. To do so, they may develop a logic model for the new intervention, develop intervention instruments, outline the material and human resources needed, set up an intersectoral coordination committee with partners, etc.

^a^Step 3 has been divided into two separate components for purposes of analysis, but was initially conceived as one step by the promoters

## Methods

### Context and research design

The work presented here is part of a larger project. The ALPS Project (*Analyse des laboratories de promotion de la santé* – Analysis of the Health Promotion Laboratory) is a multiple case study evaluating the processes and outcomes of the HPL, a professional development pilot program in public health in Quebec, Canada [35]. This paper reports the results of a descriptive study of the implementation of the HPL in a subset of the larger project (three study sites).

### Site selection and characteristics

The HPL start-up in the seven sites was spread over several months. The cases chosen for the present study were the first three sites to implement and complete the HPL program in different CSSSs (hereinafter referred to as Teams A, B and C).

### Data collection

Two sources of data were used. Implementation data were primarily collected using documentary analysis. Sources included agendas and minutes of HPL meetings (212 items), logbooks of the PHDM mentors (274 entries), internal reports (14 items), as well as educational tools (106 items) for the three sites. Because some minutes and logbook entries were missing, informal interviews with mentors were conducted to complete the information.

Data related to the characteristics of teams and their contexts were collected through the analysis of the documents mentioned above as well as through semi-structured interviews with key informants, including HPL participants, PHDM mentors, and managers and colleagues not involved in the HPL (total *N* = 28). Interviews were conducted at the end of the implementation phase. An interview grid exploring different dimensions of the laboratory experience and context (e.g. participants ‘characteristics, perceived effects and knowledge diffusion in the organization, dedicated resources and other organizational dimensions) was used to ensure consistent data collection across sites. Data pertaining to teams’ characteristics and organizational contexts were used in the present study.

Implementation profiles and possible influencing factors were presented to the three teams’ participants in a workshop that also included PHDM mentors and researchers. Validation focus groups were also held within each study site. Full ethical approvals were sought and obtained from the PHDM (#MP-ASSS-MTL-12-002) and University of Montreal (#12-094-CERES-D) ethics committees.

### Data analysis

Data pertaining to HPL activities were retrieved from the PHDM’s electronic database and paper records. Based on the HPL presentation documents and internal reports, we created a code book to define each step of the operational approach and identified activities to exemplify each step (see Additional file 1). We developed a template to facilitate coding of activities carried out at each HPL team meeting. One activity usually corresponded to one agenda point and referred to a concrete task for carrying out a particular step of the operational approach. Examples of activities include debating on a specific article (step 3) and meeting with key stakeholders to establish partnership relations in a particular field (step 6). The analytical process involved performing directed content analysis of the material in relation to the steps of the operational approach. Using this sequential content analysis, we modeled each team’s progression through the operational approach. Two of the research team members coded the data for the three teams. Coding was validated by a third research member to ensure intercoder reliability and credibility of interpretations. Coding disparities were explored and resolved by consensus among the three coders. Disagreements most often pertained to whether or not a particular agenda item ought to be characterized as an “activity” or whether a specific activity had been assigned the right code. After coding the material, we calculated the percentages of activities carried out by the different teams in each step of the program’s implementation. We examined longitudinally the pattern of activities over the whole process in each site and calculated site-specific descriptive statistics.

Coding the interview data allowed us to organize the interview content and label meaningful segments. For the purposes of this exploratory analysis, we created analytical tables on the following themes: contextual factors, characteristics of participants and HPL groups, and organizational support for learning (access to documentation and continuing education).

## Results

### Team characteristics

The three teams consisted on average of nine professionals and managers. They differed in terms of members’ activity sectors and targeted issues. Team A (occupational health; nine members) had a higher proportion of members with longer work experience, and its participants had a long history of working together. Because of their particular position in the CSSS (in response to a subregional mandate), members had a more flexible time frame that facilitated their involvement in the HPL.

Team B (student retention; ten members) consisted of participants coming mainly from the family/child/youth sector. The professionals in this sector were primarily mandated to respond to the needs of the schools of the territory and, as such, distributed their time among the different schools and the CSSS. The team members were engaged for the most part in individual practice with clients and did not have a long history of working together before the HPL.

Team C (children’s vulnerability; eight members) consisted of participants coming mainly from the CSSS’s child and family services division. The professionals were primarily mandated to offer counseling on various issues and providing ante- and postnatal support to mothers. Most members already knew each other very well before the HPL and had a long history of working together. A particularity of this site is that members ended up creating not just one, but four intervention projects acting on different factors related to protecting vulnerable children in their territory. Also of interest is the higher number of participants who withdrew (temporarily or definitively) from the HPL for various reasons—illness and maternity, among others.

Each team was guided by a different PHDM mentor, but all mentors followed the operational approach described above. All teams reported having access to informational resources and continuing education. However, many participants from sites A and B pointed out that training was limited due to budget cutbacks. While subject to the same budget restrictions, team C participants appeared to enjoy more educational opportunities because of their CSSS’s university affiliation (conferences, on-site colloquia, etc.). CSSS C also received a recognition award for its continuing education program. Table 2 summarizes the contextual and team characteristics for each site.

**Table 2:** Contextual and team characteristics

	Team A (Jan 10 – Jun 12)	Team B (Mar 10 – Mar 13)	Team C (Jan 11 – Dec 13)
Targeted issue	Occupational health	Student retention	Children experiencing vulnerability
Justification for choice of issue	Service area featured residential neighbourhoods surrounding industrial areas.	Service area had a lower average of high school completion rate than the regional average.	Service area included many immigrant families who were poorly integrated because of language barriers.
Formula	Bi-weekly meetings	Bi-weekly meetings	Bi-weekly meetings
Intervention project developed	A health promotion counseling program to support business owners who were either setting up or relocating their operations	A health promotion outreach strategy to work with schools to promote the value of education among parents	Four intervention projects: increased access to daycare facilities for marginalized families; community network to promote breastfeeding; social support project for immigrant women; community childhood–family issues table
Participants	Middle-managers (2), executive advisors (2, public health and nursing), nurses (3), community organizer (1), industrial hygienist (1), occupational health and safety physician (1)	Middle-managers (2), executive advisor on public health (1), school nurse (1), social workers (2), dental hygienist (1), community organizers (3), psychoeducator (1)	Middle-managers (2), executive advisor on public health (1), psychoeducator (1), social worker (1), nurses (2), dietitian (1), special educators (2), planning and programming officers (2)
At least 15 years of work experience	A majority of participants	About half of the participants	About half of the participants
Diplomas or work experience in public health	A few participants	No participant	A few participants
History of collaboration	Participants had a long tradition of working as a team and, for the most part, already knew each other.	Team members were engaged for the most part in individual practice with clients and had not a long history of working together before the HPL.	Most participants knew each other and had a long history of working together.
Activity sector and organizational support	Participants came mostly from the occupational health and safety division. This team has a subregional mandate assigned by the Occupational Health and Safety Commission to visit factory and businesses to monitor health risks and prevent harmful exposure for the workers. It was deemed easier for the organization to free up participants and reassign the work to others, giving the participants enough time to engage in the HPL activities.	Participants came mainly from the family/child/youth division. Professionals in this sector were primarily mandated to respond to the needs of the schools of the territory. The organization was not always able to exempt participants from their duties during the HPL because service demand was too high.	Participants came mainly from the child and family services division. Professionals were primarily mandated to offer counseling on nutrition, vaccination, education, children’s behavior and family life. They provided prenatal and postnatal support for mothers. The service demand was high and the organization did not always exempt participants from their duties during the HPL.
Participant turnover	Average	Average	High
Organizational learning culture	High access to documentation; budget cuts to continuing education	Limited access to documentation; budget cuts to continuing education	High access to documentation; university affiliation providing numerous opportunities of continuing education

### Implementation profiles

Given their particular contexts and composition, each team came up with its own adapted process for working through the operational approach.

### Descriptive statistics

Each team invested a substantial number of hours, over different time spans, in both the planning meetings and the HPL meetings themselves: Team A – 140 hours over 17 months (29 meetings); Team B – 195 hours over 26 months (40 meetings); Team C – 280 hours over 35 months (56 meetings). The core members’ participation rates were similar across the teams, ranging from 74 to 85 %.

### Number and percentage of activities related to different components

Table 3 presents percentages and absolute numbers of activities broken down by teams and by steps of the operational approach. Of interest is the large amount of activities devoted to transferring knowledge and ensuring program sustainability. Typical activities included publishing articles in internal and professional journals, giving informal and formal presentations to various internal and external audiences, and inviting colleagues and managers to attend HPL meetings. Team B conducted fewer activities of this kind, as participants waited until implementation was under way in the community before fully disseminating their experience. Steps 4 and 5—studying the problem and identifying options for actions—represented a high proportion of activities in all teams. However, inter-case differences were observed, with Team A being in a position to select its options more rapidly and Team C needing a much higher absolute number of activities to do so. Percentages of activities varied between 9 % and 19.5 % for steps 6 (develop partnerships) and 7 (implement intervention). Finally, steps 1 to 3 required small and quite similar percentages (and absolute numbers) of activities across all teams.

**Table 3:** Percentage of activities devoted to each step of the program in the three sites

Step targeted	Team A	Team B	Team C
	*N* (%)	*N* (%)	*N* (%)
*1) Identify a targeted issue and a team*	4 (2.4 %)	2 (1.4 %)	3 (1.1 %)
*2) Specify the operational approach*	11 (6.6 %)	12 (8.6 %)	12 (4.7 %)
*3a) Acquire basic concepts of public health and provide opportunities for reflection*	7 (4.2 %)	4 (2.9 %)	10 (3.8 %)
*3b) Transfer new knowledge and ensure sustainability of the program*	69 (41.6 %)	33 (23.6 %)	65 (25.3)%
4) *Study the problem (theme or targeted issue)*	28 (16.7 %)	29 (20.7 %)	29 (11.3 %)
5) *Identify options for action*	5 (3.0 %)	28 (20.0 %)	43 (16.7 %)
6) *Develop partnerships*	17 (10.2 %)	19 (13.6 %)	45 (17.5 %)
7) *Implement a new health promotion action or improve a current intervention*	25 (15.1 %)	13 (9.3 %)	50 (19.5 %)
Total	166 (100 %)	140 (100 %)	257 (100 %)
	(spanning 29 meetings)	(spanning 40 meetings)	(spanning 56 meetings)

### Patterns of activities across time

**Fig. 1:**
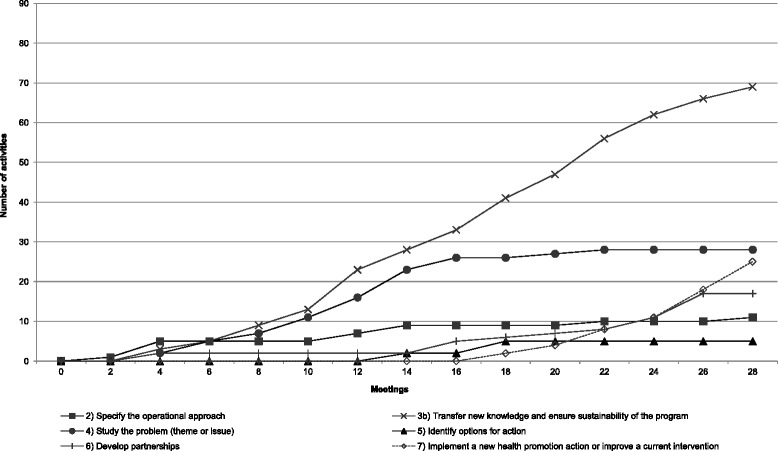
Implementation pattern of Team A’s operational approach

**Fig. 2:**
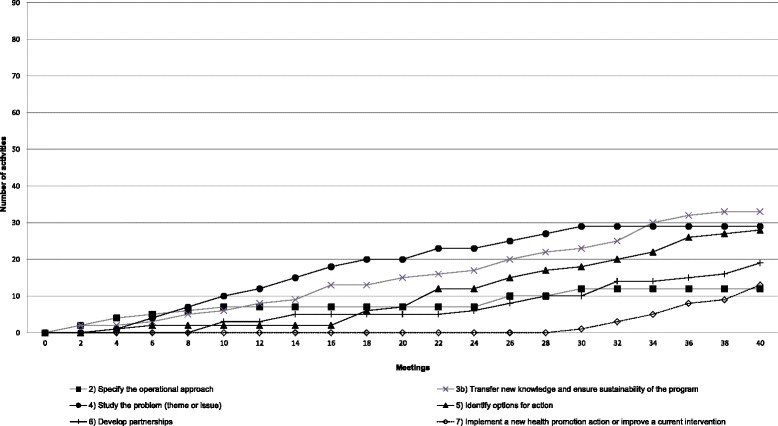
Implementation pattern of Team B’s operational approach

**Fig. 3:**
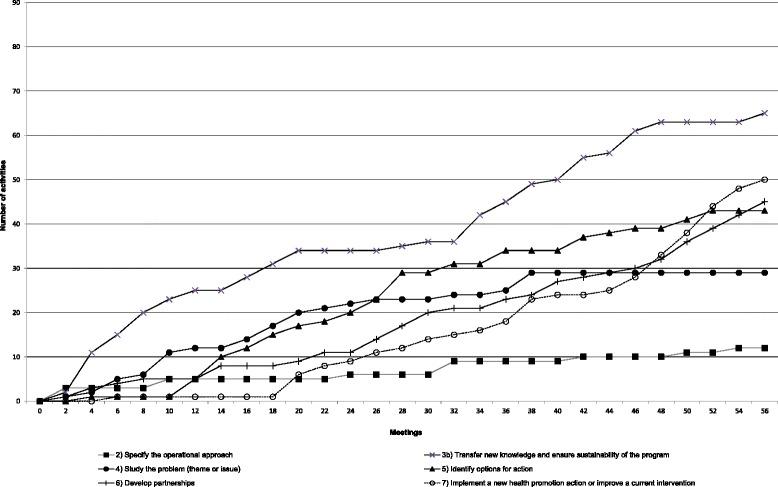
Implementation pattern of Team C’s operational approach

The HPL’s operational approach suggests an iterative process. It is possible for teams to progress through the steps either sequentially or simultaneously. As such, a team could choose to perform most of the activities in one step before initiating those in another or could make progress on several fronts simultaneously. We constructed line charts showing the cumulative numbers of activities for each step from one meeting to another. For the sake of parsimony, steps 1 and 3a, whose implementation patterns were almost identical in all teams, have been excluded from the charts. In each case, these steps were initiated early (in the first six meetings) and then remained unchanged over the course of the process. The findings showed that each team developed a unique pattern of working its way through the operational approach.

#### Team A

Team A’s work is notable in that it completed the program in much less time than did the others. Figure 1 shows that the activities conducted within step 2, which set the stage for the rest of the HPL, were implemented more frequently in the first six meetings and then sporadically repeated throughout the process. In contrast, participants engaged in activities to mobilize the organization and transfer new knowledge to colleagues in the organization (step 3b) continuously throughout the process (29 meetings). Activities dedicated to studying the issue and forging a common understanding of it (step 4) were mostly conducted in the first 16 meetings. Identifying an intervention option (step 5) took a short time, from meeting 12 to meeting 18. Finally, the team engaged in activities aimed at carrying out their project, such as developing partnerships with community actors (step 6) and implementing the intervention (step 7), mostly from the middle of the process (meeting 14) to the end.

#### Team B

Figure 2 shows a somewhat different pattern for Team B. Similarly to Team A, this team implemented activities within step 2 infrequently but regularly throughout the entire process and consistently carried out activities to transfer new knowledge to other colleagues in the organization and to ensure the program’s sustainability in the organization (step 3b). The number of these activities increased toward the end of the process (meeting 32). However, a particular feature of team B’s pattern is that the targeted issue was studied almost continuously (step 4) and options for intervention were identified regularly (step 5) throughout the process. Partnerships with local actors (step 6) were explored early on (meeting 8), and activities dedicated to implementing the team’s project (step 7) appeared relatively late (meeting 28).

#### Team C

Figure 3 reveals that team C also adopted a unique sequence of activities within the HPL. Similarly to the other teams, activities aimed at specifying the operational approach (step 2) were carried out sporadically throughout the process. Activities devoted to transferring knowledge and mobilizing the organization (step 3b) were sustained consistently throughout the process. A particular feature of this team’s work was the development and concurrent implementation of four projects (instead of one) within the HPL. Thus, activities geared to the development of these projects (i.e., activities related to steps 4, 5, 6 and 7) were implemented almost continuously from the beginning to the end of the process.

### Comparative analysis and identification of potential influencing factors

The results showed that the first three steps required small and relatively similar percentages of activities for all three teams. Thus, the implementation of activities related to identifying the issue, specifying the operational approach, and acquiring basic concepts of public health did not seem much affected by the particular contexts of teams and participants. However, the picture that emerged for the other steps was quite different. For example, Team C took much longer to complete the operational approach, in particular with regard to identifying options, developing partnerships, and implementing action. In comparison, Team A took half the time to complete the HPL, for example, devoting only five activities to identifying an option for action (the preferred intervention).

Many factors may have contributed to this differential implementation profile (see Table 2). Team C’s lengthier process may have been due to the fact that they elected to work on four projects rather than one, or to their higher rates of extended absenteeism (e.g. pregnancy leave). Moreover, participants in Teams B and C were both working in a context of high demand for individual services to clients, often without being replaced, resulting in a high workload and possibly less availability for the HPL work. In contrast, while they experienced some modification of membership over the course of the process, participants of Team A reported having a more flexible work schedule, which may have facilitated their involvement. They also selected a project whose actions were closely linked to their current practices, whereas Teams B and C chose projects that deviated more in this respect. Lastly, two factors may have slowed down progress in team B: a less advantageous profile than the other sites in term of 1) indicators of organizational learning culture; and 2) familiarity between participants at the start of the HPL.

Validation sessions conducted with participants confirmed the accuracy of the implementation profiles generated. Some participants provided further details regarding the project developed within their laboratory, which enabled researchers to flesh out the descriptions. Many positive comments were made, in particular with regard to the program’s operational approach and the freedom granted in the choice of the health promotion project and the pace of its development.

## Discussion

The findings presented here show that a health promotion professional development program aimed at local public health organizations can be successfully tailored to different settings without compromising its core components. In this paper, we present a program that allows for two types of adaptation: (1) initial **pre-formatting to accommodate organizational context** (as shown in the case descriptions); and (2) **ongoing adaptation of implementation to take into account the needs and contexts of the participants** (as shown in the results). The HPL program’s original parameters were tailored to the implementation sites by allowing each CSSS to choose a target issue based on its needs and the availability of its staff. Then the program’s operational approach was implemented in ways that allowed for ongoing adaptation to the teams’ particular contexts and needs, leaving room for flexibility and creativity in carrying out the different steps. Even though each team developed a unique pattern of implementing the activities, the results of the analysis show that every step of the program was implemented. These steps, which are crucial to the entire change process intended by the program, represent the ‘active ingredients’ of the HPL, its core components.

The implementation of core components is meant to ensure a program’s fidelity to its logic model and theory, thereby promoting achievement of the intended outcomes [10, 36]. However, as discussed at length by Morrison et al. [17], the needs and constraints of implementation sites often “place bounds on how an intervention can be implemented” (p. 134). Uncontrolled variations in the intervention’s implementation can threaten the program’s integrity to the point where it might no longer be reasonable to expect the hypothesized outcomes across sites. In the present pilot project, training and follow-up meetings with the PHDM mentors most likely helped participants to not deviate extensively from the proposed operational approach while allowing sufficient flexibility to respond to each teams’ objectives and needs. Future replications of this approach in similar organizations but without the support of the original promoters will shed light on the extent to which adherence can be maintained with less monitoring. The difficulty of stating definitively which aspects of a component (content, frequency, delivery mode) are crucial to achieve the intended outcomes has already been highlighted [17]. “Some initial consensus on how to define a component is needed. Content focused on the constructs of the theory guiding the intervention may be a wise starting point” (p. 137) [17]. In this particular project, the HPL operational approach was indeed a useful tool in this respect.

Another issue concerns adapting the context to the intervention. In fact, most often success depends not only on tailoring the intervention to the implementation context, but also on **adjusting the local context** to optimize the implementation [16, 37]. The implementation science literature has emphasized both “the need to adapt interventions to the service context and to adapt aspects of outer context (i.e., service system) and inner context (i.e., organization) to effectively implement [evidence-based programs]” (p. 33) [16]. In the cases presented here, for instance, the CSSSs agreed to free up HPL participants during working hours to attend meetings. They were also open to accommodating the new health promotion practices developed by the team. Results showed, however, that in practice it was not always possible to exempt participants from their duties, because of human resource shortages and lack of replacement options. This situation may have contributed to slowing down the process in sites B and C. However, explanatory analysis of other factors also highlighted a possible role for history of collaboration and dimensions of organizational learning culture. Indeed, in Site C, a favorable profile with respect to those two dimensions might have helped ensure the HPL’s success even in the presence of factors likely to influence the process negatively.

To be successful, professional development programs in public health and health promotion must be adaptable to practitioners’ complex real-world practice settings while preserving core program components. This implementation analysis has shown that the HPL clearly satisfies this criterion. Capturing the heterogeneity of forms the intervention may take, as was done here, will make possible in-depth impact analyses, including the testing of program–context interactions to identify program outcomes predictors. Such work is essential to advance knowledge on the action mechanisms of professional development programs.

### Limitations and future avenues of research

This study may suffer from certain limitations, and the results should be interpreted accordingly. A first limitation was that the implementation steps were assessed and compared across teams using numbers of activities rather than the amount of time devoted to each, which could be misleading, as the time required for different activities can vary. We believe activity numbers provide a generally accurate picture of the amount of effort dedicated to achieving one particular step, and this assumption was also deemed valid by the HPL participants. Nevertheless, we suggest that a qualitative analysis of activities, taking into account the time devoted to each, would be a useful complement to consider in future research. Second, some documentary sources, such as mentors’ logbooks and minutes of meetings, were not homogeneous and varied from one team to another. To overcome this limitation, several data sources were used to provide a comprehensive picture of each team’s activities. Third, at some meetings, teams took few or no minutes and some mentors did not keep detailed notes of the activities discussed. To address this challenge, missing data were completed through informal interviews with key informants. Lastly, our analysis of the contextual factors affecting the implementation process was exploratory and limited to a few organizational dimensions, setting aside other key dimensions related to, for example, the external context, the PHDM mentors, and other organizational-level factors [38–40]. Future research should not only build upon existing theoretical models and other conceptual contributions in implementation science to ensure these dimensions are taken into account, but should also use a design that will facilitate investigation of the complex interplay between these factors.

Notwithstanding these limitations, we employed several means to ensure the validity of this study’s conclusions. Most of the researchers were involved with the PHDM program team for several years to develop a deep, comprehensive understanding of the HPL model and its operational approach. The findings were also validated with PHDM mentors and participants to ensure accuracy of data and interpretation of results. We believe these results provide a useful picture of the HPL implementation process in the three sites and, more specifically, of the HPL’s capacity to be adapted to different contexts while preserving its core components. In the next phase of our research, we will investigate the relationships between these different implementation profiles and program outcomes.

## Conclusions

In this article we have explored the question of implementation fidelity and adaptation of a professional development program in health promotion. Based on our findings, we have proposed some points to consider when developing and implementing such a program. First, this study has highlighted the need to make explicit the core elements of program theory that are critical to achieving the program’s intended outcomes. This component analysis can be done in reference to a theory, a proven logic model, or evidence-based science, but should make it possible to identify the features of the program that account for its outcomes. Second, our results emphasize the importance of providing flexibility in a program’s design, such that it can be adapted beforehand to different contexts and then on the ground as the implementation unfolds. Third, we have explored the potential role of organizational dimensions in the implementation process. Given the great variety of public health and health promotion practice settings, the heterogeneity of professional backgrounds, and the complexity of determinants that need to be addressed to promote the health of populations, program adaptability is a prerequisite for health promotion professional development. The present study has confirmed the great potential of the HPL in this respect.

## Additional file

Additional file 1:
**Analysis grid for activities conducted in the laboratories (code book).**
