# Supplementary material for: An analysis of the adaptability of a professional development program in public health: results from the ALPS Study
Source: BMC Health Serv Res. 2015 Jun 14;15:233. doi: 10.1186/s12913-015-0903-3 (PMC4465469; doi:10.1186/s12913-015-0903-3)
Supplement: Additional file 1: — Analysis grid for activities conducted in the laboratories (code book). [file 12913_2015_903_MOESM1_ESM.pdf]

Analysis grid for activities conducted in the laboratories (code book)

Name of process component	Objective of the component	Examples of activities related to the component
(1) Identify an issue and a team	To present to laboratory to the CSSSs and the teams and to set the parameters for the laboratory in the CSSS (team and issue).	Presenting the laboratory (history, general model, reasons for the project in the CSSS) to the management and the team involved.<br><br>Selecting a team interested in the project.<br><br>Selecting an issue to serve as the initial theme for the laboratory.
(2) Determine the operational process	To present plan, and adjust the laboratory's operational approach to the team.	Presenting the operational approach.<br><br>Identifying, through discussions, actions that will help minimize constraints and optimize incentives to participate in the laboratory.<br><br>Presenting and discussing the roles of the different actors in the laboratory.<br><br>Setting up the logistics of the project and determining how the laboratory will function (e.g. discussing the order in which the different components of the process will be addressed; deciding on certain functional details, such as minute taking; taking stock of progress and considering future prospects for the laboratory, as appropriate at different points in the operational process).
(3) Acquire basic concepts in public health	To facilitate participants' understanding of key concepts in public health and health promotion.	Reading and discussing (reading club) texts on public health (determinants of health, definition of health promotion).<br><br>Reading about and discussing the population-based approach.<br><br>Doing exercises to acquire key public health concepts
(4) Make the laboratory known and mobilize the extended team, the	To describe and explain the laboratory within the CSSS and to other audiences, and to foster commitment from other teams	Developing a communications plan to make the laboratory known in the CSSS and elsewhere.

CSSS management and other external organizations and teams	and from the CSSS for both the laboratory and the project.	Writing articles on the laboratories for in-house newsletters<br><br>Making presentations on the project at team meetings and other events (e.g. “Labs among us”, conferences, presentations to other teams in the CSSS, management, inter-CSSS or inter-laboratory meetings). Following up on those presentations as needed.<br><br>Making the laboratory known outside and encouraging exchange (e.g. discussion forums, external colleagues invited to the laboratory as guests).
(5) Explore the issue more deeply	To develop a shared understanding of the issue, learn more about it, and determine how and from what angle the issue will be tackled.	Discussing the angle from which to act (or the determinant to act upon) to address the issue or to determine which issue to focus on (if there are several).<br><br>Interpreting data on the health status of the population in the territory to support the selection of a specific issue.<br><br>Collecting, analyzing and interpreting supplementary data to support the angle from which the specific issue is to be addressed.<br><br>Developing a shared understanding of the issue and its determinants (e.g. readings and discussions about the issue, reading club).<br><br>Engaging in priority-setting activities to select the angle from which the issue will be addressed (e.g. list of criteria for selecting the issue or angle, priority grid).
(6) Identify possibilities for action	To determine what health promotion intervention (project) will be developed to act upon the issue, depending on the angle chosen. Also called “outlining the issue”.	Discussing relevant health promotion interventions (strategies used, determinants involved, changes targeted).<br><br>Discussing new ways of working that are focused on health promotion and on social determinants (could include readings and discussions on this topic, reading club).

		Developing an inventory of the professionals' current practices with regard to the issue (e.g. a practice profile).<br><br>Deciding collectively on the action to be developed in relation to the issue identified.
(7) Develop a partnership	To develop a clear understanding of the concept of partnership among participants, to identify potential partners for the project, and to plan a partnership with other (internal) teams or other (external) organizations or actors.	Undergoing general and advanced training on working in partnership (may include readings and discussions on partnership, a reading club).<br><br>Discussing the advantages and disadvantages of sector-based vs. partnership-based action.<br><br>Identifying key partners.<br><br>Identifying action strategies to mobilize potential partners around the project.<br><br>Defining the desired partnership in relation to the issue being addressed.
(8) Implement a new health promotion intervention	To plan, after the issue has been selected, the implementation and coordination of the project developed in the laboratory.	Making explicit the components of the intervention.<br><br>Developing a logic model for the intervention.<br><br>Designing tools for the intervention, a communications plan for the project, and a pilot project.<br><br>Setting up an intersectoral coordinating committee with the partners.
(9) Evaluate the laboratory	To evaluate the laboratory's activities, in whatever way is possible and for a variety of purposes.	Holding an information session on the subject of evaluating the laboratory<br><br>Conducting focus groups and other evaluations with the participants facilitated by an evaluator.<br><br>Participants evaluating the laboratory (performance assessment), e.g. to produce an internal status report or to improve the laboratory formula.

		Conducting informal evaluations of sessions (satisfaction levels).<br><br>Surveying participants through questionnaires.<br><br>Presenting consent forms.
(10) Other types of activities	Activities that do not fit within any other component of the operational approach.	Becoming informed on other issues that will not have been taken into consideration (developing literacy).

Glossary

Angle: The aspect or determining factor of the issue upon which we wish to act by means of a health promotion intervention. Also specific issue.

Issue: The particular problem (e.g. workplace health, school retention) upon which the laboratory teams have chosen to work.

Team: Consists of all the regular and occasional participants in the laboratory at any particular meeting.

Extended or sectoral team: Consists of all the laboratory participants as well as the other professionals of the teams from which the participants have been drawn (e.g. the workplace health team: a small number of professionals from the workplace health team were selected to take part in the laboratory, but the extended team includes all professionals assigned to workplace health in the CSSS, not only the participants).

Participants: Includes all types of laboratory participants, i.e., the CSSS health professionals, managers and team leaders, external participants (students, experts), and PHDM mentors.

Project: Health promotion intervention developed in the laboratory.

Laboratory meetings: As opposed to the meetings held to prepare the laboratory (preparatory meetings), the laboratory meetings are 3-hour sessions in which the team engages in laboratory activities. Also called implementation meetings when the project is in the implementation stage.
